# Machine Learning Methods to Personalize Persuasive Strategies in mHealth Interventions That Promote Physical Activity: Scoping Review and Categorization Overview

**DOI:** 10.2196/47774

**Published:** 2024-11-15

**Authors:** Annette Brons, Shihan Wang, Bart Visser, Ben Kröse, Sander Bakkes, Remco Veltkamp

**Affiliations:** 1 Digital Life Center Amsterdam University of Applied Sciences Amsterdam Netherlands; 2 Department of Information and Computing Sciences Utrecht University Utrecht Netherlands; 3 Centre of Expertise Urban Vitality Amsterdam University of Applied Sciences Amsterdam Netherlands; 4 Department of Computer Science University of Amsterdam Amsterdam Netherlands

**Keywords:** artificial intelligence, exercise, mobile app, adaptive, tailoring, supervised learning, reinforcement learning, recommender system

## Abstract

**Background:**

Although physical activity (PA) has positive effects on health and well-being, physical inactivity is a worldwide problem. Mobile health interventions have been shown to be effective in promoting PA. Personalizing persuasive strategies improves intervention success and can be conducted using machine learning (ML). For PA, several studies have addressed personalized persuasive strategies without ML, whereas others have included personalization using ML without focusing on persuasive strategies. An overview of studies discussing ML to personalize persuasive strategies in PA-promoting interventions and corresponding categorizations could be helpful for such interventions to be designed in the future but is still missing.

**Objective:**

First, we aimed to provide an overview of implemented ML techniques to personalize persuasive strategies in mobile health interventions promoting PA. Moreover, we aimed to present a categorization overview as a starting point for applying ML techniques in this field.

**Methods:**

A scoping review was conducted based on the framework by Arksey and O’Malley and the PRISMA-ScR (Preferred Reporting Items for Systematic Reviews and Meta-Analyses extension for Scoping Reviews) criteria. Scopus, Web of Science, and PubMed were searched for studies that included ML to personalize persuasive strategies in interventions promoting PA. Papers were screened using the ASReview software. From the included papers, categorized by the research project they belonged to, we extracted data regarding general study information, target group, PA intervention, implemented technology, and study details. On the basis of the analysis of these data, a categorization overview was given.

**Results:**

In total, 40 papers belonging to 27 different projects were included. These papers could be categorized in 4 groups based on their dimension of personalization. Then, for each dimension, 1 or 2 persuasive strategy categories were found together with a type of ML. The overview resulted in a categorization consisting of 3 levels: dimension of personalization, persuasive strategy, and type of ML. When personalizing the timing of the messages, most projects implemented reinforcement learning to personalize the timing of reminders and supervised learning (SL) to personalize the timing of feedback, monitoring, and goal-setting messages. Regarding the content of the messages, most projects implemented SL to personalize PA suggestions and feedback or educational messages. For personalizing PA suggestions, SL can be implemented either alone or combined with a recommender system. Finally, reinforcement learning was mostly used to personalize the type of feedback messages.

**Conclusions:**

The overview of all implemented persuasive strategies and their corresponding ML methods is insightful for this interdisciplinary field. Moreover, it led to a categorization overview that provides insights into the design and development of personalized persuasive strategies to promote PA. In future papers, the categorization overview might be expanded with additional layers to specify ML methods or additional dimensions of personalization and persuasive strategies.

## Introduction

### Background and Related Work

Regular physical activity (PA) decreases the risk of several diseases, improves quality of life and well-being, and improves mental health. Despite these positive effects, physical inactivity is a serious and still growing worldwide problem. Worldwide, 28% of adults and 81% of adolescents are insufficiently physically active. They do not meet the World Health Organization recommendations of at least 150 minutes per week of moderately intense PA for adults and 60 minutes of moderate PA per day for adolescents [1].

Mobile health (mHealth) interventions have been shown to be effective in promoting PA [2-5]. Intervention success can be improved by implementing effective persuasive strategies, also called behavior change techniques, such as goal setting, monitoring, rewards, reminders, education, activity suggestions, and feedback [6-8]. Moreover, personalized mHealth interventions are more effective in promoting PA than nonpersonalized interventions [9-12]. In mHealth, personalization means adapting intervention strategies to individual characteristics such as gender, disease, coping strategy, current location, current PA level, or performed PA [13,14]. The combination of success factors for personalization and for persuasive strategies can take place on several dimensions, such as the type of feedback, content of the messages, timing of the messages, rewards, and personal settings [9,11,15].

To personalize persuasive health-promoting interventions, machine learning (ML) has become increasingly popular in recent years [16,17]. ML is an important subfield of artificial intelligence, with numerous applications in domains such as robotics, natural language processing, and computer vision. The key methods of ML include supervised learning (SL), unsupervised learning (UL), and reinforcement learning (RL). Several kinds of ML techniques have been used in adaptive interventions that promote PA. SL, in which the prediction is based on labeled data, can be used for classification to predict a category and for regression to predict a quantity. SL for classification, for instance, was used in a chatbot-based digital coach to improve PA levels [18] and a mobile app with personalized feedback to promote PA in cardiovascular disease rehabilitation [19]. SL was used for regression to predict low blood sugar levels in patients with diabetes based on their previous blood glucose levels and performed PA [20]. On the other hand, UL analyzes unlabeled data and can be implemented to cluster data or build a recommender system (RS). An RS provides suggestions that fit the user’s needs or preferences. Clustering, for example, was applied in a PA advisor system to increase PA [21], whereas an RS was developed to recommend adequate educational content to users to improve their knowledge regarding PA and a healthy diet [22]. Finally, there is RL, which is an ML method based on rewarding desired actions. RL is often used to personalize the timing of messages, also called a just-in-time adaptive intervention [23]. To choose the appropriate RL algorithm, problems are categorized as Markov decision processes (MDPs) or as multi-armed bandit (MAB) and contextual bandit problems. MDPs can model decision-making problems when the results are partly random and partly controlled by the user. MDP-based RL, for example, was used in an adaptive intervention to personalize motivational coaching messages to promote healthy behavior such as PA [24]. MAB is used when a series of choices has to be made without knowing personal preferences and the consequences of a choice. MAB, for instance, was the basis of an adaptive personalized messaging smartphone app to improve PA levels [25].

There are many studies [5,26-32] that show an overview of the effectiveness of mHealth interventions on PA, each focusing on different target groups and outcomes. Several recent reviews [9,11,32-36] have zoomed in on personalizing persuasive interventions, but none of them have focused on personalization with the use of ML. On the other hand, some reviews [16,37,38] have addressed the use of ML to personalize PA-promoting interventions more generally but have not studied the combination of ML and personalizing persuasive strategies. To start with, Chaudhari et al [37] reviewed personalized PA interventions using ML and other methods but focused on personalizing the timing of PA-promoting messages instead of other persuasive strategies such as content of messages or level of difficulty. In addition, Oyebode et al [38] and Goh et al [16] studied ML methods to personalize interventions for health and well-being—including PA—in which persuasive strategies were implemented but did not focus either on the ML methods used to personalize these persuasive strategies.

### Objectives and Contribution

To the best of our knowledge, no review has addressed personalizing persuasive strategies using ML in the field of promoting PA yet. Therefore, insights into the characteristics of studies that did implement ML to personalize persuasive PA-promoting strategies and an indication of missing information is already a contribution to this field. Moreover, considering the interdisciplinary nature of this field, it would be helpful for interventions to be designed and developed in the future to have some guidance in choosing the best-fitting ML technique considering the persuasive strategies. Designers and researchers in the field of persuasive strategies lack such an insightful overview. Therefore, the first objective of our study was to provide an overview of implemented persuasive strategies and their corresponding implemented ML techniques in this field. We conducted a scoping review to reach this goal as this is a useful approach to analyze knowledge gaps and identify key characteristics of studies [39]. Moreover, we aimed to categorize these findings and build a categorization overview as a starting point for applying ML techniques to personalize persuasive strategies in PA-promoting interventions.

## Methods

### Overview

A scoping review was conducted following the PRISMA-ScR (Preferred Reporting Items for Systematic Reviews and Meta-Analyses extension for Scoping Reviews) criteria [40]. The PRISMA-ScR checklist is provided in Multimedia Appendix 1. The scoping review was conducted using the framework by Arksey and O’Malley [41], later extended by Levac et al [42], which consists of the following five stages: (1) identifying the research question; (2) identifying relevant studies; (3) selecting studies; (4) charting the data; and (5) collating, summarizing, and reporting the results. In addition to these steps, we provide a categorization overview based on the results of the scoping review.

### Step 1: Identifying the Research Question

Corresponding to the 2 objectives, our research questions were defined as follows: (1) What ML techniques have been used to personalize persuasive strategies in mHealth interventions that promote PA? (2) How to guide future designers, developers, and researchers in choosing ML techniques to personalize persuasive strategies in PA-promoting interventions?

### Step 2: Identifying Relevant Studies

We searched for studies published until February 24, 2023, in 3 databases: Scopus, Web of Science, and PubMed. These databases were searched because they cover relevant studies in the digital health domain. To find relevant papers for answering the research questions, all studies should match the following semantics: *digital persuasive interventions* *promoting PA* that are *personalized* to participants’ needs through *machine learning* techniques. We searched for these concepts in the title, abstract, and keywords. The exact query that was run on all databases can be found in Multimedia Appendix 2. In addition, the reference lists of included papers and relevant review papers were searched by hand. These papers were categorized as *manual* in the identification section of the PRISMA-ScR flow diagram.

### Step 3: Selecting the Studies

After the initial search, duplicates were removed. The remaining articles were screened by 2 reviewers (AB and SW) in 2 phases. First, titles and abstracts were screened using the ASReview software [43,44]. Studies meeting at least one of the exclusion criteria (Textbox 1) were removed. ASReview uses ML to efficiently screen large amounts of titles and abstracts [45]. Moreover, the studies are only reviewed based on content rather than irrelevant metadata such as author and journal names as only titles and abstracts are shown [46]. Both reviewers started with the same set of papers and had to choose at least 2 papers to label before screening to initially train the algorithm. Moreover, the algorithm improves itself based on the labels of the reviewer. Thus, the order of papers shown for screening differs between the reviewers as it depends on both the reviewers’ choice of initial papers to label and the labels given during the screening process. On the basis of several relevant and irrelevant studies labeled by the reviewers, a model is trained to sort the remaining studies by order of relevance. When labeling all studies, the model constantly improves itself. Thus, both reviewers did manually screen all titles and abstracts, but because of the order of relevance, it was more efficient than the usual method in a random order.

**Inclusion and exclusion criteria.**

**Inclusion criteria**

- Articles: peer-reviewed articles, conference proceedings, or book chapters on qualitative, quantitative, and mixed method studies or protocols
- Language: English
- Application: digital intervention, either stand-alone or hybrid with a health care provider, with at least one personalized persuasive strategy
- Machine learning (ML) technique: supervised ML, unsupervised ML, or reinforcement learning
- Health behavior promoted by the intervention: physical activity (including walking, running, cycling, performing exercises, and performing sports activities) either alone or as part of several health behaviors

**Exclusion criteria**

- Articles: conference abstracts, reviews, letters, editorials, comments, non–peer-reviewed articles or book chapters, white papers, or articles with no full text available
- Language: all other languages
- Application: nonadaptive intervention or persuasive strategy not specified
- ML technique: rule-based if-then systems, predefined formulas, manual personalization, ML method not specified, computer vision, natural language processing, or robotics
- Health behavior promoted by the intervention: health behaviors other than physical activity

Next, the full texts of all the remaining studies were reviewed. Studies meeting all the inclusion criteria (Textbox 1) were included. When both reviewers did not agree, a third reviewer (SB) was asked for the final decision. Both reviewing rounds were conducted blinded, meaning that both reviewers had no access to each other’s findings before completing the full task. A dataset consisting of the screened papers together with the reason for exclusion can be found here [47]. Afterward, all the included papers were categorized based on the projects they belonged to. As project names, we used the name of the intervention or distinctive key terms included in the study. For each project that included several papers, the paper with the most detailed information regarding the implemented persuasive strategies and ML method was labeled as the main paper. From the Target Group subheading in the Results section onward, we only use this main paper as a reference to keep the report clear.

### Step 4: Charting Data

Data extraction was performed by one reviewer (AB) and verified by a second reviewer (SW). The extracted data were recorded in a Microsoft Excel form that was tested by 2 reviewers (AB and SW) beforehand. We extracted data regarding general information of the paper, target group, PA intervention, and the implemented technology. A detailed overview of the extracted information is shown in Textbox 2.

**Extracted data from the full texts.**

**General**

- Title
- Authors
- Year of publication
- Type of paper (journal article, conference proceeding, or book chapter)
- Objective
- Main results

**Intervention target group**

- Adults or children
- Healthy or with disabilities

**Intervention**

- Description of the intervention
- Persuasive strategy
- Physical activity (PA) application field
- Underlying theories
- Stand-alone or hybrid with human interaction

**Technology**

- Machine learning (ML) category and method
- ML technology
- Gamification or not
- Types of data as input for algorithm
- Features used for adaptation
- Dimension of personalization
- Platform

**Study**

- Study type
- Real users or simulation
- Outcomes regarding PA or ML technique

### Step 5: Collating, Summarizing, and Reporting the Results

We presented an overview of the literature search using the PRISMA-ScR flow diagram. Moreover, we analyzed and described the extracted data from the included papers. We focused on the developed intervention, target group, implemented ML method, dimension of personalization, underlying persuasive strategy, type of study, and main results. Furthermore, we summarize the underlying psychological theories and some technical details of the developed interventions.

### Building the Categorization Overview

On the basis of the resulting overview of implemented ML methods to personalize persuasive strategies in PA-promoting interventions, we provided a categorization overview. This consists of 3 levels, starting with the dimension of personalization. The persuasive strategies are shown per dimension together with the ML method that was implemented in most of the projects. In addition, we show the projects that support the highlighted ML method for that specific combination of persuasive strategy and dimension of personalization.

The ML methods shown in the categorization overview are based on the number of projects that implemented that ML method for a specific persuasive strategy within a dimension of personalization. As the outcome measures of all the included projects differed widely and not all projects had published results yet, we did not consider the results of the included projects in the categorization overview.

## Results

### Overview of Literature Search

**Figure 1:**
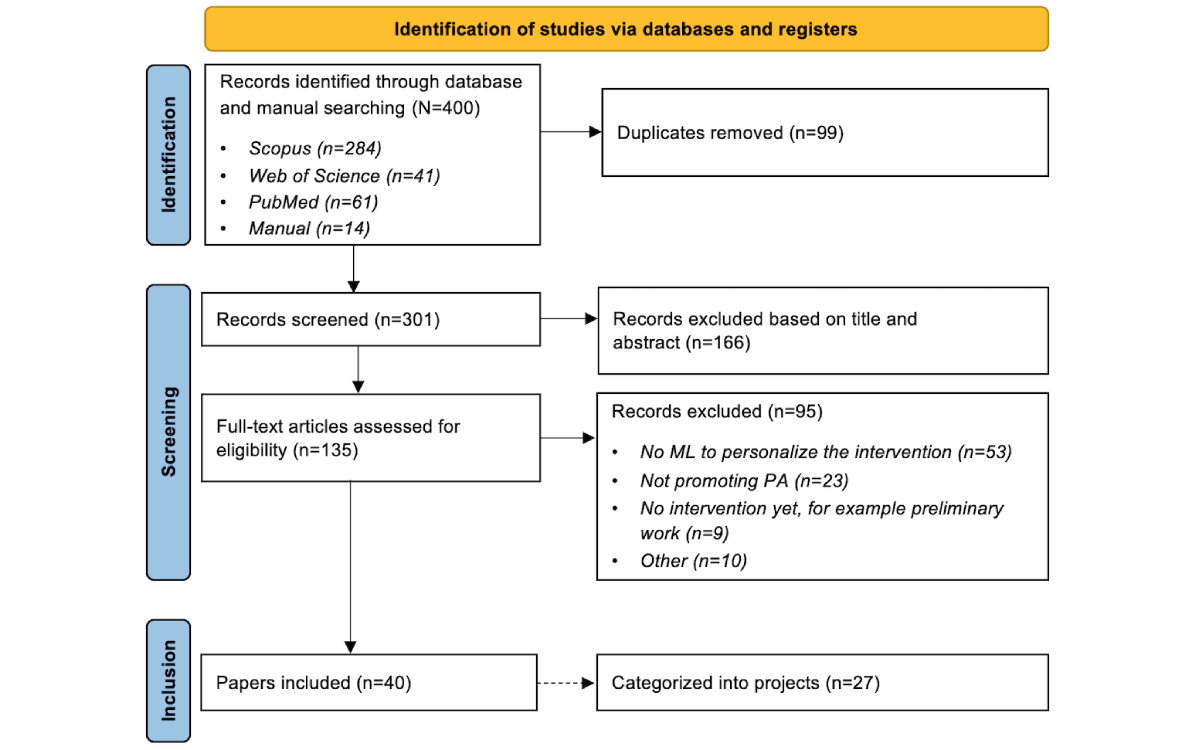
PRISMA-ScR (Preferred Reporting Items for Systematic Reviews and Meta-Analyses extension for Scoping Reviews) flow diagram. ML: machine learning; PA: physical activity.

The search query yielded 400 papers in total. These studies were scanned for duplicates, which resulted in a total of 75.3% (301/400) of unique papers. The abstracts of the unique papers were assessed against the exclusion criteria, resulting in a shortlist of 44.9% (135/301) of the papers. Main reasons for excluding abstracts were no promotion of PA, no personalized intervention, no use of ML to personalize the intervention, and review papers. Reviewing the full texts of these 135 shortlisted papers resulted in 40 (29.6%) included papers, being 13.3% (40/301) of the screened unique papers and belonging to 27 projects. Most excluded papers (53/95, 56%) discussed interventions that were personalized without using ML. A lot of the other papers were removed because the intervention did not promote PA (23/95, 24%) or there was no intervention yet (9/95, 9%). The PRISMA-ScR flow diagram with the aforementioned numbers is shown in Figure 1.

### Overview of Included Projects

In total, 40 papers that used ML to personalize persuasive strategies in PA-promoting interventions were included. These papers belonged to 27 projects. All projects and their corresponding papers are shown in Table 1 categorized by the dimension of personalization. Project Power2DM was the only intervention that belonged to 2 dimensions of personalization and, therefore, is included twice to give a complete overview of each dimension.

**Table 1:** Overview of all the included papers categorized by the dimension of personalization. In the Relevant papers column, the main article is mentioned first.

Project name		Relevant papers	Intervention target group	Persuasive strategy	ML^a^ category and method	ML technique	Features used for adaptation	Study type	Study outcome
**Dimension of personalization: timing of messages**									
	Heartsteps	Tomkins et al [48] and Liao et al [49]	Adults with hypertension	Reminders	RL^b^: contextual bandit	IntelligentPooling	Performed PA^c^, location, weather, day of the week, and current time	Simulation (to improve algorithm) and pilot	All participants except 1 improved their step count with the intervention; 26% improvement in step count in simulations; IntelligentPooling algorithm performed better than Thompson sampling algorithm
	Power2DM^d^	Gönül et al [24] and Glachs et al [50]	Adults with diabetes	Reminders (timing), motivational messages, and feedback (type of feedback)	RL: MDP^e^ and contextual bandit	Customized version of eligibility traces	Time, location, PA status, phone screen status, emotional status, and number of reminders and motivational messages that have been sent	Simulation	Number of required messages to achieve the goal was lower for RL MDP than for general RL
	PAUL	Wang et al [51,52] and Sporrel et al [53]	Adults	Reminders	RL: MDP	Policy gradient RL (REINFORCE algorithm)	Performed PA, current time, and calendar	Feasibility study	83.3% of reminders provoked PA within 50 min; 66.7% of PAs were performed within 5 hours after the reminder
	Ally	Mishra et al [18], Kramer et al [54], and Künzler et al [55]	Adults	Goal setting and monitoring	SL^f^: classification	Logistic regression	Date, time, battery level of phone, device interaction, and PA	Simulation and pilot	Higher receptivity in ML group than in the controls (simulation); participants who were more receptive were more likely to achieve their goals (pilot)
	U4Fit	Pilloni et al [56]	Adults	Feedback and monitoring	SL: classification	RF^g^	Covered distance, workout duration, rest time, average speed, burned calories, and time elapsed since previous workout	Simulation	In 70% of cases, the ML intervention correctly predicted whether a sportsman would stop exercising and need feedback or motivational messages
**Dimension of personalization: content of messages**									
	Quantified Self	Erdeniz et al [57]	Adults	PA suggestion	SL: classification; UL^h^: recommender system	K-nearest neighbor; collaborative filtering	Age, gender, location, chronic diseases, oxygen saturation, heart rate, and performed PA	Pilot	≥75% of PA recommendations were accurate
	WellHealth	Lo et al [58]	Adults with chronic neck or back pain	PA suggestion	SL: classification	Neural network	Present condition, history of condition, past investigation, social history, drug history, and 24-hour PA pattern	Observational study	Increased time spent on therapeutic exercises
	MyBehavior	Rabbi et al [59,60]	Both healthy adults and adults with obesity	PA suggestion	RL: multi-armed bandit; UL: clustering	EXP3^i^; BIRCH^j^ online clustering algorithm	Current location, performed PA, and PA behavior pattern	RCT^k^	Significantly (*P*=.05) better improvement in PA in ML-personalized intervention compared to nonpersonalized intervention
	Mining Minds	Banos et al [61]	Adults	PA suggestion	SL: classification; UL: recommender system	SVM^l^ and Gaussian mixture modeling; content-based filtering	Accelerometer data and feedback from users given on recommendations to improve the system	Pilot test	Average response time after recommendation was between 3 and 40 min
	PA Advisor	Li et al [21]	Adults	PA suggestion	SL: classification; UL: clustering	DT^m^, SVM, and RF; hierarchical clustering with Gower distance	Location, PA, and general physiological characteristics	Design	No results yet
	Pro-Fit	Dharia et al [62]	Adults	PA suggestion	SL: classification; UL: recommender system	Gradient boosting and DT; collaborative filtering	Accelerometer data, feedback from users on given recommendations, and user profile	Simulation (to improve algorithm) and user study	Most participants felt that the ML-personalized intervention was successful in motivating them to increase PA levels
	WalkPal	Sansrimahachai [63]	Older adults	PA suggestion	SL: classification	Neural network	Heart rate, location, and walking statistics	Simulation	Reasonable prediction accuracy of exercise min with a mean average error of –17 to +17 weekly exercise min
	Blockchain	Jamil et al [64]	Both healthy adults and adults with obesity	PA suggestion	SL: classification	SVM	BMI, performed PA, and user activity	Simulation	SVM was the best-performing ML method with an accuracy of 92%
	PA Recommendation	Zhao et al [65]	Adults	PA suggestion	SL: classification; UL: recommender system	SVM; collaborative filtering	Age, purpose of exercise, vegetarian or not, wake-up time, video game preference, workout with trainer or not, gender, BMI, favorite type of musing during exercise, and favorite sports brand	Simulation and pilot	Prediction accuracy of ≥70% in simulation
	Wireless Health	Suh et al [66]	Adults	PA suggestion, feedback, and social comparison	SL: classification (personalized activity); UL: recommender system (feedback and social comparison)	DT; collaborative filtering	Heart rate, accelerometer data, pressure, and gyroscope data	Pilot	Exercises were performed more accurately, and participants reported increase in motivation; increase of 25.54% in calories burned compared to nonpersonalized training protocol
	selfBACK	Mork and Bach [67] and Sandal et al [68]	Adults with chronic low back pain	PA suggestion and education	SL: classification	Case-based reasoning	Demographics, medication, quality of life score, sleep, mood, stress, pain, and PA	Design	No results yet
	CoCare	Cerón-Ríos et al [22]	Both healthy adults and adults with obesity	Education	SL: classification; UL: recommender system	DT; collaborative filtering	Current location, indoor location, friends, date, and daily schedule	Feasibility study	90% of recommendations were accurate
	drBart	Pelle et al [69]	Adults with osteoarthritis	Goal setting	UL: recommender system	Contextual multi-armed bandit approach	Gender, age, height, weight, symptoms, quality of sleep, and maximum walking distance	Design	No results yet
	HNGW	Dijkhuis et al [70]	Adults	Feedback	SL: classification	RF	Steps	Pilot test	RF was the best-performing ML method with an accuracy of 93% and an *F*_1_-score of 0.9
	Healthy Behavior Messages	Kadri et al [71]	Adults	Feedback	SL: classification	DT	Accelerometer data	Simulation	DT performed better than the BiLSTM^n^ classifier with an *F*_1_-score of 62%
	MedFit	Prabhu et al [19]	Adults	Feedback	SL: classification	SVM	Accelerometer data	Design	No results yet
	BeWell+	Lane et al [72]	Adults	Feedback	SL: classification	Naïve Bayes	PA	Design	Intervention can operate successfully on consumer smartphones, and users understand the personalized feedback and respond by taking steps
**Dimension of personalization: type of messages**									
	Diabetes Messages	Hochberg et al [73] and Yom-Tov et al [74]	Adults with type 2 diabetes	Feedback	RL: contextual bandit	Linear regression with Bolzmann sampling	Demographics, PA, and reaction to messages	Pilot	Significant difference (*P*=.04)between personalized and nonpersonalized version: increase in PA in the ML-personalized intervention and decrease in PA in the nonpersonalized intervention
	Personalization Paradox	Zhu et al [75]	Adults	Feedback, monitoring, and social comparison	RL: multi-armed bandit	—^o^	Performed PA and self-reported motivation	User study	No significant difference in step count between ML-personalized intervention and nonpersonalized intervention; significant difference (*P*=.004) in motivation between ML-personalized intervention and nonpersonalized intervention
	Diamante	Aguilera et al [25] and Figueroa et al [76-78]	Adults	Feedback	RL: multi-armed bandit	Thompson sampling	Not discussed	Design	No results yet
	Power2DM^d^	Gönül et al [24] and Glachs et al [50]	Adults with diabetes	Reminders (timing), motivational messages, and feedback (type of message)	RL: MDP and contextual bandit	Customized version of eligibility traces	Time, location, PA status, phone screen status, emotional status, and number of interventions sent for planned activity	Simulation	RL MDP performed better than standard RL; number of required messages to achieve the goal was lower for RL MDP than for general RL
**Dimension of personalization: level of difficulty of PA**									
	Maze VR	Huber et al [79]	Adults	PA that fit the capabilities	RL: MDP	Deep RL	ECG^p^ and Borg scale	Proof of concept	Prototype was able to adapt both the cognitive and physical difficulty of the game for participants who were unsatisfied with a very easy level; the algorithm helped keep participants in a state of flow
	Pathologys	Aguilar et al [80]	Adults	PA that fit the capabilities	SL: classification	DT and neural network	Heart rate and performance	Pilot	Neural network had greater impact on progress and motivation than DT

^a^ML: machine learning.

^b^RL: reinforcement learning.

^c^PA: physical activity.

^d^Indicates that the project was included in the table twice because it belonged to 2 dimensions of personalization.

^e^MDP: Markov decision process.

^f^SL: supervised learning.

^g^RF: random forest.

^h^UL: unsupervised learning.

^i^EXP3: Exponential-weight algorithm for Exploration and Exploitation.

^j^BIRCH: Balanced Iterative Reducing and Clustering Using Hierarchies.

^k^RCT: randomized controlled trial.

^l^SVM: support vector machine.

^m^DT: decision tree.

^n^BiLSTM: bidirectional long short-term memory.

^o^Not applicable.

^p^ECG: electrocardiogram.

### Projects

Project Heartsteps [48,49] developed a digital coach to improve the PA levels of patients with hypertension by providing PA reminders. Project PAUL [51-53] developed a digital coach that provided PA reminders as well in addition to several other nonpersonalized persuasive strategies. The digital coaches of projects Diabetes Messages [73,74], Diamante [25,76-78], BeWell+ [72], MedFit [19], Healthy Behavior Messages [71], and HNGW [70] all provided personalized feedback to increase PA levels in general, all having their own target group. The digital coach of project Ally [18,54,55] provided personalized goal-setting and monitoring messages. Project U4Fit [56] did not design a digital coach itself but developed an algorithm that predicted whether a sportsman would stop exercising so that their human coach could contact them.

In total, 41% (11/27) of the projects suggested personalized PAs. A total of 55% (6/11) of them focused on a PA plan or schedule, whereas 45% (5/11) of them included specific exercises or activities. The Quantified Self project [57], Mining Minds [61], PA Advisor [21], and PA Recommendation [65] all suggested personalized activities to promote PA in general, the latter focusing on PA recommendations for exergames. Projects WalkPal [63], Pro-Fit [62], and Wireless Health [58] all focused on training. They included a walking exercise plan [63], fitness schedule [62], and interval training schedule [66]. Wireless Health also provided personalized music recommendations and social comparison to motivate users to achieve the proposed training schedule. Both MyBehavior [59,60] and Blockchain [64] combined the suggested PAs with a dietary plan to increase PA and decrease calorie intake. In WellHealth [63] and selfBACK [67,68], the activity schedule was part of a self-management plan for people with chronic low back pain. The first focused on adherence to exercise therapy, whereas the aim of the second project was to prevent chronic pain.

The drBart project [69] focused on self-management as well by providing personal goals. This project aimed to optimize nonsurgical care. Project CoCare [22] suggested personalized multimedia content about PA and a healthy diet. Project Personalization Paradox [75] attempted to increase PA motivation by providing social comparison. Both Maze VR [79] and Pathologys [80] developed exergames with dynamic difficulty adjustment to promote PA. Finally, project Power2DM [24,50] personalized the timing and frequency of motivational coaching messages to promote healthy behavior, including PA.

### Target Group

All 27 projects designed adaptive PA-promoting interventions for adults. In total, 26% (7/27) of them were designed for adults with a disease, such as cardiovascular disease [19,48], diabetes [24,74], osteoarthritis [69], and chronic neck or back pain [58,67]. A total of 4% (1/27) of the projects were designed for older adults specifically [63]. In total, 59% (16/27) of the interventions were designed for adults in general [18,21,25,51,56,57,61,62,65,66,70-72,75,79,80], and 11% (3/27) of the interventions had both healthy adults and adults with obesity as their target groups [22,60,64].

### Persuasive Strategies

In total, 4 different dimensions of personalization were found in all projects. Except for 4% (1/27) of the projects [24], all projects included only 1 dimension of personalization. Most of the projects (17/27, 63%) personalized the content of the messages [19,21,22,57,58,60-67,69-72]. A total of 19% (5/27) of the projects focused on the timing of the messages [18,24,48,51,56], whereas 15% (4/27) of the projects personalized the type of feedback in the messages [24,25,74,75]. For instance, this regarded positive or negative feedback and feedback in which someone’s results were compared to someone else’s or to their own previous results. In addition, 7% (2/27) of the projects personalized the level of difficulty of PAs [79,80], meaning that the difficulty of PAs was aligned with the capabilities of the participants.

In total, 9 different personalized persuasive strategies were incorporated into the developed interventions: reminders, goal setting, monitoring, motivational messages, feedback, social comparison, PA suggestions, education, and PA that fits capabilities. Most projects that personalized the timing of messages (3/5, 60%) included reminders [24,48,51]. Moreover, goal setting [18], monitoring [18,56], and feedback [56] were applied in this group. Most projects that personalized the content of messages (11/17, 65%) used PA suggestions as a persuasive strategy [21,57,58,60-67]. Of those projects, 18% (2/11) also personalized feedback and used social comparison [66] and education [67]. Feedback was personalized in another 15% (4/27) of the projects [19,70-72], and education was the persuasive strategy in another project [22]. The remaining project focused on personalized goal setting [69]. All projects that personalized the type of messages (4/27, 15%) included feedback as a persuasive strategy [24,25,74,75]. In addition, they included motivational messages [24], monitoring, and social comparison [68]. The 7% (2/27) of the projects that personalized the difficulty of PA used PA suggestions that fit the participants’ capabilities as a persuasive strategy [79,80]. This means that, for instance, easier PAs were suggested when previous activities appeared to be too hard for the participants.

### ML Methods

The ML methods that were implemented in the PA-promoting interventions can be divided into 3 categories: RL, SL, and UL. A variety of methods was used within these categories. To start with, 30% (8/27) of the projects used RL. These projects regarded personalized timing of reminders [24,48,49,51], personalized feedback types [24,25,74,75], personalized PA suggestions [60], and personalized difficulty of PAs [79]. Most (6/8, 86%) used RL based on contextual bandit or MAB problems [24,25,48,60,74,75]. MDP was the basis for the remaining projects that implemented RL algorithms to personalize their persuasive strategies [24,51,79]. One of the projects combined both strategies [24]. Except for project Personalization Paradox, all projects reported the specific RL technique that was used to personalize the persuasive strategies. All projects implemented other specific RL techniques: customized version of eligibility traces [24], policy gradient RL [51], deep RL [79], IntelligentPooling [48], Exponential-weight algorithm for Exploration and Exploitation (EXP3) [60], Thompson sampling [25], and linear regression with Bolzmann sampling [74].

SL was implemented in 56% (15/27) of the projects, and they all used SL for classification. These projects included personalized timing of monitoring and feedback messages [18,56]; personalized level of difficulty of PA [80]; and personalizing the content of the messages, including personalized PA suggestions [57,58,61-67], education [22,67], and feedback [19,67,70-72]. A variety of techniques was applied in the group that used SL: logistic regression [18], random forest (RF) [56,70], neural networks (NNs) [58,63,80], k-nearest neighbor [57], decision tree (DT) [22,62,66,71,80], naïve Bayes [72], case-based reasoning [67], and support vector machine [19,61,64,65]. A total of 19% (5/27) of the projects, which applied SL, combined this with UL for RS [22,57,61,62,66]. Except for 1, all of them personalized the content of the messages. Most (4/5, 80%) used collaborative filtering [22,57,62,66], whereas one project used content-based filtering [61]. In addition, 7% (2/27) of the projects combined SL with UL for clustering using hierarchical clustering with Gower distance [21] and Balanced Iterative Reducing and Clustering Using Hierarchies online clustering [60]. Only 4% (1/27) of the projects implemented UL for RS without SL [69]. They used a contextual MAB approach to personalize goal setting.

### Study Outcomes

A total of 22% (6/27) of the projects had PA-related outcomes as the main study results. In total, 67% (4/6) of these projects compared the PA-related outcome between an ML-personalized intervention and a nonpersonalized intervention [60,66,74,75]. In 75% (3/4) of these projects, the ML-personalized version performed better in terms of performed PA than the nonpersonalized version [60,66,74]. This difference was reported to be significant with respectively *P*=.05 [60] and *P*=.04 [74] in 67% (2/3) of the projects. No significant difference in PA levels was found in the remaining project, although a significant difference (*P*=.004) in motivation was found in favor of the ML-personalized intervention [75]. In addition, 15% (4/27) of the projects found positive effects regarding PA when using the ML-personalized intervention without comparison to a nonpersonalized intervention. Improved step count [48], increased time spent on exercises [58], and successful motivation of participants to increase PA levels [62] were reported. Finally, 4% (1/27) of the projects reported that personalizing the difficulty of PAs in an exergame helped keep participants in a state of flow [79].

In total, 33% (9/27) of the projects reported outcomes regarding their developed algorithm as the main study results. A total of 56% (5/9) of these projects focused on the accuracy of their classifications. Accurate PA recommendations with 75% [57], 70% [65], and 92% [64] accuracy were reported, as well as an accuracy of 90% for predicting personalized educational content [22] and 70% for predicting the need for motivational feedback [56]. Moreover, an accuracy of 93% for predicting the probability of achieving a daily step goal [55] and an *F*_1_-score of 62% for predicting the performed PAs [71] were found. In addition, 11% (1/9) of the projects reported a mean average error of –17 to +17 minutes per week on the prediction of performed exercise [63]. A total of 22% (2/9) of the projects reported the response time after a message. The average time of performing PA after receiving a recommendation was between 3 and 40 minutes in one of the projects [61], whereas 66.7% of the PAs were performed within 5 hours after the reminder in another project [51]. The latter also reported that 83.3% of the reminders provoked PA.

Only 19% (5/27) of the projects compared specific ML techniques, all comparing techniques from the same type of ML. One study reported that the number of messages needed to achieve a goal was lower for the MDP RL algorithm than for general RL algorithms [24]. In total, 40% (2/5) of the projects compared DT with another classifier. DT was preferred over bidirectional long short-term memory (BiLSTM) in one of those projects [71], whereas NNs achieved better results in the other project [80]. DT, logistic regression, support vector machine, and k-nearest neighbor were compared in 40% (2/5) of the projects. In the first project, DT performed best [64]. In the second project, adaptive boosting, NNs, stochastic gradient descent, and RF were added to the comparison, and RF performed best [70].

Of the 21 projects that reported results, 16 (76%) achieved this with real users [18,22,48,51,57,58,60-62,65,66,70,74,75,79,80], whereas the remaining 5 (24%) did so in simulations [24,56,63,64,71]. A total of 11% (3/27) of the projects did describe the design and a protocol for evaluation, but no results had been published yet [25,67,69]. In total, 7% (2/27) of the projects described the design, but no evaluation plan was found [19,21]. One project did evaluate the intervention, but no outcome regarding PA or the developed algorithm was discussed [72].

### Other Findings

Only 30% (8/27) of the projects [19,24,51,57,60,67,69,75] reported the underlying theories that supported the choice of their implemented persuasive strategies. Most mentioned behavior change techniques as the basis for their intervention. Although not all studies reported specific behavior change techniques, the Fogg Behavior Model [81]; the Capability, Opportunity, and Motivation–Behavior (COM-B) model [82]; and self-determination theory [83,84] were mentioned several times. In addition, the social cognitive theory [85], the theory of social comparison by Buunk [86], and the theory of planned behavior [87] were included.

Most interventions (16/27, 59%) were designed as mobile phone apps. Only 11% (3/27) of the projects developed a web application. Except for 4% (1/27) of the projects, no human interaction, for instance, with a health care professional, was implemented. The project that did choose a hybrid version instead of a stand-alone digital intervention enabled contact with a human sports coach [56]. A total of 3 of the interventions included gamification elements, of which 2 (67%) developed an exergame with personalized difficulty of PA in the game. Regarding the input features for the ML algorithms, most were based on sensor data such as performed PA, heart rate, and location. In addition, phone log information, questionnaire results, and personal characteristics were used as input features.

### Categorization Overview

In the included projects, we searched for differences and similarities in several characteristics: the dimension of personalization, implemented persuasive strategies, intervention target group, ML category and method, ML technique, and study outcome. Clear categories regarding the dimension of personalization and overlapping approaches regarding persuasive strategies and ML methods were found, whereas no categories could be established for the target group, study type, and study outcome. Regarding the target group, not enough differences were found to split the projects into categories. On the other hand, regarding the study outcome and ML technique, too many differences were found to propose categories at this level.

**Figure 2:**
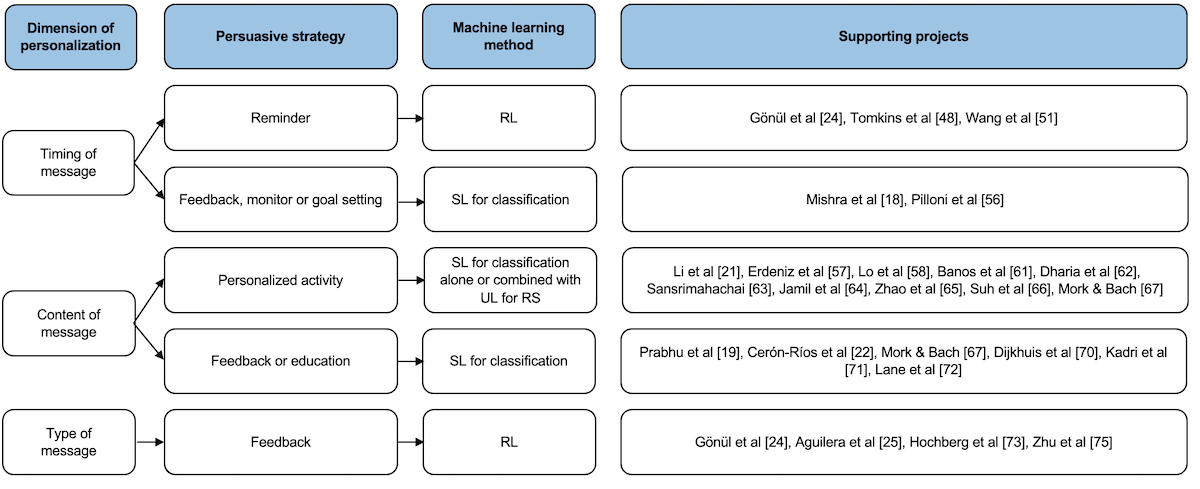
Proposed categorization overview as a starting point for choosing machine learning methods when implementing adaptive persuasive strategies in mobile health interventions that promote physical activity [18,19,21,22,24,25,48,51,56-58,61-67,70-73,75]. RL: reinforcement learning; RS: recommender system; SL: supervised learning; UL: unsupervised learning.

Thus, our categorization overview, shown in Figure 2 [17,18,20-23,45,48,51,56-58,61-68,71,72,75], consists of 3 levels: dimension of personalization, persuasive strategy, and ML method. For the first level, 4 dimensions of personalization were found in the included projects: the timing, content, and type of messages and the level of difficulty of PA. As only 7% (2/27) of the projects personalized the level of difficulty of PA [79,80] and both implemented another type of ML, there was not enough support to include this dimension of personalization in our framework. Therefore, the dimension of personalization level consists of timing, content, and type of messages.

For the timing of messages, the persuasive strategies were divided into 2 groups: the timing of reminders and the timing of messages regarding feedback, monitoring, and goal setting. RL was implemented in the 3 projects in this category that personalized the timing of reminders [24,48,51]. SL for classification was used in both projects [18,56] that personalized the timing of messages with feedback, monitoring, or goal-setting information. The difference in methods between the timing of reminders and that of feedback, monitoring, and goal-setting information might be explained by the idea that reminders often need to be sent at a specific time or time frame related to a lot of activities, whereas feedback, monitoring, and goal-setting messages are often sent at a time frame only related to the performed PA. The latter kind of messages should be sent, for instance, during, directly after, or 15 minutes after performing PA, which can be translated to 3 categories and, therefore, is suitable for solving using SL for classification. On the other hand, reminders for PA should be sent, for example, after 2 hours of inactivity on Tuesday mornings, 10 minutes after taking the bus on Thursday evenings, and also at 8:05 AM on Saturdays. RL is suitable to solve such specific timing problems. Moreover, with RL, the algorithm can be improved over time with additional information, such as the time between the reminder and the actual performed PA. This can be done without training the whole dataset again, which would have been the case when using SL.

Regarding personalizing the content of the messages, 2 groups of persuasive strategies were found. For personalizing both PA suggestions and feedback or educational messages, SL for classification was used in most projects (15/17, 88%). To personalize PA suggestions, either SL or SL combined with UL for RS was implemented most often. This category is supported by 82% (9/11) of the projects in this group, which performed SL [57,58,61-67], of which 44% (4/9) combined SL for classification with UL for RS [57,61,62,65]. SL for classification can be used to predict which activities are the best fit to the participants’ needs and characteristics, such as age, current PA level, or disease. When there is, for instance, a large number of PAs that can be suggested, SL can be combined with RS to improve the recommendations over time based on feedback from the user regarding the quality of the recommendation. The predictions that are performed using SL can then be used as a starting point. Regarding personalizing the content of feedback or educational messages, most projects in this category (6/8, 75%) only implemented SL [19,22,67,70-72]. For example, this can be used to predict which educational topics or feedback messages regarding the performed PA are suitable. The number of options for these types of messages is often much smaller than the number of possible PA suggestions. As RSs are particularly useful in selecting personalized items from a large dataset, improvement of the results using an RS is less suitable for personalizing feedback messages or educational topics than for personalizing PA suggestions. For this reason, and because only 17% (1/6) of the projects in this group combined SL with RS [22], we did not include this combination in our categorization.

Regarding the personalization of the type of messages, only feedback was found as a persuasive strategy. To personalize the type of feedback messages, all 4 projects implemented RL [24,25,74,75]. This, for instance, can be used to predict whether a participant’s motivation is best improved using positive, neutral, or negative feedback. When RL is implemented, the algorithm can be improved over time without retraining or relabeling.

## Discussion

### Principal Findings

The objective of our study was to provide an overview of ML methods used to personalize persuasive strategies in PA-promoting interventions and present a categorization overview based on these findings as a starting point for implementing ML methods in this field. We analyzed the details of the developed PA interventions, the implemented personalized persuasive strategies, the ML methods used, and the results of 40 studies belonging to 27 projects. These papers could be categorized based on the dimensions of personalization, which resulted in 4 groups. For each dimension, 1 or 2 persuasive strategy categories were found and linked to ML methods.

On the basis of these included papers and dimensions found, we provided a categorization overview, now consisting of three layers: (1) the dimension of personalization, (2) the personalized persuasive strategy, and (3) the ML method. To personalize the timing of reminders, RL was mostly implemented, whereas SL for classification was implemented most often for personalizing the timing of messages regarding feedback, monitoring, and goal setting. For personalizing the content of PA suggestions and feedback or educational messages, most projects (15/17, 88%) implemented SL for classification as well. Regarding personalized PA suggestions, this was implemented either alone or combined with UL for RS. Finally, RL was often implemented to personalize the type of feedback messages. The categorization overview can be a starting point in using ML to personalize persuasive strategies in PA-promoting mHealth interventions.

### Strengths, Limitations, and Opportunities

A strength of our study is the systematic analysis of multiple mHealth interventions using personalized persuasive strategies with ML to promote PA. We analyzed the persuasive strategies and ML methods of 27 projects on several levels. Inherent to conducting a scoping review, we did not consider the quality of the studies, which could have led to biased results. Moreover, we initially intended to only include projects that had published results. Although all these projects showed positive results on their own outcome measures, we were not able to compare the results because the outcome measures differed widely. Therefore, we decided to also include projects that had not published results yet. This additional information was in line with the projects that had published results and, therefore, resulted in a stronger support for the presented categorization overview. However, it is a limitation that this categorization overview could only be based on the number of interventions in which ML methods were implemented and that effectiveness and performance differences were not considered. When more projects publish their results in the future, this could lead to enough comparable information regarding study outcomes so that recommendations based on the effectiveness of particular ML methods can be made. Moreover, challenges faced, such as data availability, interpretability, privacy concerns, and performance differences between data collection and labeling approaches, could then be discussed. Other aspects that might be elaborated more on in the future when more information is published are the underlying theories that support the choice of implemented persuasive strategies and the use of human contact to personalize persuasive strategies in health care settings. It is remarkable that, for now, only 30% (8/27) of the projects described such underlying theories and only 4% (1/27) of the projects implemented the possibility to have contact with a human being.

The need for more studies publishing performance or effectiveness to compare study outcomes was also discussed in the studies of Goh et al [16] and Chaudhari et al [37]. Goh et al [16] performed a scoping review on ML techniques to personalize interventions for health and well-being, including PA, and mentioned that more studies should examine interventions in a more mature, developmental stage to appraise the impacts of such interventions more prudently and confidently. Chaudhari et al [37] only studied personalized timing of PA-promoting messages and also included personalization without ML. However, they also discussed that more studies evaluating the effectiveness of the interventions are required to learn which aspects of personalization are promising.

Although we have carefully formulated our inclusion criteria and constructed our search strategy, such restrictions always result in excluding interesting papers and information. For future research, it might be interesting to explore how ML techniques have been used to personalize persuasive strategies specifically. Moreover, the search could be extended to more specific databases regarding health care and behavior change. To widen the perspective, it might also be compelling to compare ML methods and persuasive strategies in PA-promoting interventions with those in interventions that focus on other domains of health promotion.

Because we used ASReview, we could efficiently screen a lot of papers, and we were able to focus on the content rather than on irrelevant metadata. A strength of our study is the dataset with all labels and reasons for exclusion. This improves the reproducibility of our study, and this information is useful for future meta-reviews. However, a limitation is that the trained ASReview models could not be extracted from the ASReview program.

The proposed categorization overview now has 3 levels: the dimension of personalization, persuasive strategy, and type of ML. As the objective of our study was to provide a categorization overview as a starting point for implementing ML to personalize persuasive strategies in PA-promoting interventions, we chose to present the dimension of personalization as the first level. Adding a level on top of this to guide in choosing personalized persuasive strategies is outside the scope of our study. A lot of work has already been published in this field, for instance, studies that address the implementation of effective persuasive strategies [6-8] and the effect of personalized interventions [9-12]. At first, we wanted to add a fourth level to our categorization overview to further specify the ML methods by recommending specific ML techniques. However, not enough overlapping techniques were found in the included projects to support this information for all dimensions of personalization. The only ML technique that had enough support was collaborative filtering as UL method for an RS to personalize the content of PA suggestions. The number of choices per level was defined by the analyzed projects. We found 3 different dimensions of personalization with enough support and, for each dimension, 1 or 2 persuasive strategies with several supporting projects. Remarkably, these 3 dimensions concerned messages. This might indicate that messages are useful for personalization. A possible explanation might be that contact with the participant is the basis of a lot of persuasive strategies. In an mHealth intervention, such contact is often replaced by digital messages.

The current categorization overview might already be helpful for future designers, developers, and researchers that plan to implement ML for personalizing persuasive strategies in PA-promoting interventions. However, the categorization overview is expected to be extended when the results of more interventions are published. For instance, more choices per level with new dimensions of personalization, such as personalizing the level of difficulty of PA, or persuasive strategies could be added. Moreover, a fourth level might then be added to specify ML methods by categorizing ML techniques. It would be interesting to study whether specific ML techniques can be linked to features such as the kind of data used for personalization, the target group, the specific intervention goal, or the underlying psychological theories. In addition, it would be interesting to explore whether large language models will be used more often to personalize the content of messages now that generative artificial intelligence has become available to the public and has recently shown promising results.

Oyebode et al [38] conducted a review on ML techniques in personalized systems for health and well-being. Although they did not focus on persuasive strategies, they did discuss PA-promoting interventions specifically. Our results are in line with theirs, although we included 40 papers belonging to 27 projects, whereas they included 8 papers regarding PA. In addition, they gave some recommendations. These regarded general implementation challenges, such as data quality– and infrastructure-related issues. When designing new personalized persuasive PA-promoting interventions, it might be helpful to combine our categorization overview with their recommendations. This leads us to another important strength of our study: the provided categorization overview. We did not only show an insightful overview of ML methods used but also categorized this information as a starting point for the design and development of ML-personalized PA interventions.

### Conclusions

Our categorization overview framework links 3 dimensions of personalization and several persuasive strategies to implemented ML methods. Regarding the timing of messages, RL was implemented most often to personalize the timing of reminders, and SL was implemented to personalize the timing of feedback, monitoring, and goal-setting messages. When personalizing the content of messages, most projects implemented either SL or both SL and RS for PA suggestions and SL for feedback or educational messages. When personalizing the type of feedback messages, most projects implemented RL algorithms. The provided categorization overview can be used as a starting point in the design and development of personalized persuasive strategies to promote PA. When more of such mHealth intervention results are published in the future, the categorization overview might be expanded with specific ML techniques or with additional dimensions of personalization and persuasive strategies, such as personalizing the level of difficulty of PA. Moreover, when more results regarding performance or effectiveness are published, recommendations might be given on which ML methods to implement.

## Appendix

Multimedia Appendix 1 PRISMA-ScR (Preferred Reporting Items for Systematic Reviews and Meta-Analyses extension for Scoping Reviews) checklist.

Multimedia Appendix 2 Search query.

## Abbreviations

BiLSTM: bidirectional long short-term memory
COM-B: Capability, Opportunity, and Motivation–Behavior
DT: decision tree
EXP3: Exponential-weight algorithm for Exploration and Exploitation
MAB: multi-armed bandit
MDP: Markov decision process
mHealth: mobile health
ML: machine learning
NN: neural network
PA: physical activity
PRISMA-ScR: Preferred Reporting Items for Systematic Reviews and Meta-Analyses extension for Scoping Reviews
RF: random forest
RL: reinforcement learning
RS: recommender system
SL: supervised learning
UL: unsupervised learning
