# Supplementary material for: Machine Learning Methods to Personalize Persuasive Strategies in mHealth Interventions That Promote Physical Activity: Scoping Review and Categorization Overview
Source: J Med Internet Res. 2024 Nov 15;26:e47774. doi: 10.2196/47774 (PMC11607567; doi:10.2196/47774)
Supplement: Multimedia Appendix 2 [file jmir_v26i1e47774_app2.pdf]

Multmedia Appendix 2 – Search query

Concept	Relevant keywords	In query
Digital persuasive intervention	health intervention; ehealth; mhealth; uhealth; health feedback; digital tool; mobile application; persuasive; persuasion	“health intervention” OR ehealth OR mhealth OR uhealth OR “health feedback” OR “digital tool” “mobile application” OR “persua*”
Promoting PA	Physical activity; exercise; exercising; sport; sports; walk; walking	“physical activity”; exercis*; sport; walk
Personalized	Personalized; personalization; personalised; personalisation; tailored; tailoring; adaptive; adapted; adapt; just in time; just-in-time; dynamic difficulty adjustment;	“personali*” OR “tailor*” OR “adapt*” OR “just in time” OR “dynamic difficulty adjustment”
Machine learning	Articifial intelligence; machine learning; gamification; gamificating; data mining; supervised learning; decision tree; naïve bayes, k-nearest neighbour, support vector machine, decision tree, random forest, unsupervised learning; clustering; recommender system; reinforcement learning; recommender system; recommendation system; Markov decision process; MDP; partially observable markov decision process; POMDP; multi armed bandit; contextual bandit	“artificial intelligence” OR “machine learning” OR “gamificat* OR “data mining” OR “supervised learning” OR “decision tree” OR “naïve bayes” OR “k nearest neighbo*” OR “knn” OR “support vector machine” OR “svm” OR “decision tree” OR “random forest” OR “unsupervised learning” OR “clustering” OR “recommend* system” OR “markov” OR “MDP” OR “POMDP”OR “bandit”

Final query

TITLE-ABS-KEY=(("artificial intelligence” OR “machine learning" OR “gamificat*” OR “data mining” OR "supervised learning" OR “decision tree” OR “naïve bayes” OR k nearest neighbo*” OR “knn” OR support vector machine” OR “svm” OR “decition tree” OR “random forest” OR "unsupervised learning" OR “clustering” OR “recommend* system” OR "reinforcement learning" OR “Markov” OR “MDP” OR “POMDP” OR “bandit”) AND ("personali*" OR "tailor*" OR "adapt*" OR "just in time" OR “dynamic difficulty adjustment”) AND ("physical activity" OR exercise OR sport OR walk) AND("health intervention" OR ehealth OR mhealth OR uhealth OR "health feedback" OR "digital tool*" OR "persua*" OR "mobile application"))
